# Seasonal quadrivalent mRNA vaccine prevents and mitigates influenza infection

**DOI:** 10.1038/s41541-023-00752-5

**Published:** 2023-10-12

**Authors:** Christina M. Kackos, Jennifer DeBeauchamp, Christopher J. H. Davitt, Jan Lonzaric, Robert E. Sealy, Julia L. Hurwitz, Marcelo M. Samsa, Richard J. Webby

**Affiliations:** 1 Department of Infectious Diseases, St. Jude Children’s Research Hospital, Memphis, TN USA; 2 St. Jude Children’s Research Hospital Graduate School of Biomedical Sciences, Memphis, TN USA; 3 GreenLight Biosciences, Woburn, MA USA; 4 Present Address: BostonGene, Waltham, MA USA

**Keywords:** RNA vaccines, Influenza virus

## Abstract

Annually, seasonal influenza is responsible for millions of infections and hundreds of thousands of deaths. The current method for managing influenza is vaccination using a standardized amount of the influenza virus’ primary surface antigen, hemagglutinin (HA), as the intended target of the immune response. This vaccination strategy results in vaccines with variable efficacy year to year due to antigenic drift of HA, which can be further exacerbated by manufacturing processes optimizing growth of vaccine virus in eggs. Due to these limitations, alternative vaccine platforms are actively being explored to improve influenza vaccine efficacy, including cell-based, recombinant protein, and mRNA vaccines. mRNA’s rapid, in vitro production makes it an appealing platform for influenza vaccination, and the success of SARS-CoV-2 mRNA vaccines in the clinic has encouraged the development of mRNA vaccines for other pathogens. Here, the immunogenicity and protective efficacy of a quadrivalent mRNA vaccine encoding HA from four seasonal influenza viruses, A/California/07/2009 (H1N1), A/Hong Kong/4801/2014 (H3N2), B/Brisbane/60/2008 (B-Victoria lineage), and B/Phuket/3073/2013 (B-Yamagata lineage), was evaluated. In mice, a 120 μg total dose of this quadrivalent mRNA vaccine induced robust antibody titers against each subtype that were commensurate with titers when each antigen was administered alone. Following A/California/04/2009 challenge, mice were fully protected from morbidity and mortality, even at doses as low as 1 μg of each antigen. Additionally, a single administration of 10 μg of quadrivalent mRNA was sufficient to prevent weight loss caused by A/California/04/2009. These results support the promise of this mRNA vaccine for prevention and mitigation of influenza vaccine.

## Introduction

Influenza is a globally impactful pathogen that causes excess morbidity and mortality in humans through annual epidemics and sporadic pandemics^1^. Annually, seasonal influenza infection is estimated to result in 291,000–650,000 deaths worldwide^2^. In addition to the human cost of influenza, the virus is also responsible for widespread economic losses^3^. To protect against this widespread respiratory pathogen, the first influenza vaccine was developed in the 1940s by harvesting, concentrating, and inactivating virus from the allantoic fluid of embryonated hens’ eggs. Vaccination continues to be the primary defense employed against influenza, with the majority of vaccine used globally employing manufacturing processes that have changed little with time^4,5^.

Three types of influenza vaccine are currently available within the United States: inactivated influenza vaccines (IIV), live attenuated influenza vaccines (LAIV), and recombinant hemagglutinin (HA) protein subunit vaccines^6^. These vaccines induce immune responses that primarily target the HA protein of a representative strain from each of the four influenza subtypes endemic in humans: A(H1N1), A(H3N2), B-Victoria lineage, and B-Yamagata lineage^4^. While HA is highly immunogenic, it also undergoes constant antigenic drift. This necessitates formulation meetings to assess needs for updating the vaccines to reflect contemporary viruses in circulation. In addition to antigenic drift, the means by which most IIV and LAIV are produced provide additional opportunities for immune escape by influenza viruses^7^. The time-consuming manufacture and distribution of influenza vaccines require that vaccine strain selection occur over six months before vaccine use, creating a window in which a new variant may emerge or become unexpectedly dominant^8^. This can result in mismatch between one or more of the vaccine components and circulating strains, as seen by the A(H3N2) mismatch in the 2015–2016 Northern Hemisphere flu season^9^. Further complicating this issue is the method by which vaccine virus stocks are grown. The majority of IIV and LAIV are produced by growing influenza virus in embryonated hens’ eggs, an environment distinct from the mammalian cell, in which the virus can acquire growth-promoting mutations^7^. These egg adaptations may affect viral antigenicity and create further antigenic distance between the vaccine and circulating strains. Cell-based manufacture has been expanded in recent years, but this technology has its own set of challenges and is therefore not as widespread as egg-based manufacture and does not shorten the time from strain selection to vaccine administration substantially^10^. The combination of these factors results in vaccines that have unpredictable efficacy from year to year.

mRNA vaccines have been under development for decades, but the COVID-19 pandemic has catapulted this technology into the clinic. mRNA vaccines for influenza have several advantages over traditional IIV and LAIV. Production of mRNA vaccines is a fully in vitro process without the use of eggs or cell culture, eliminating the risk of viral growth-promoting mutations that may affect antigenicity. This is a particular concern for H3N2 vaccination as egg-adapted vaccine strains require a mutation that alters a key antigenic site^11^. Indeed, vaccination with H3 mRNA has been shown to more effectively induce antibodies against contemporary H3N2 viruses than IIV^12^. mRNA vaccines can also be produced more quickly than IIV and LAIV, which may reduce the time from strain selection to clinical administration and allow for a more rapid response to future influenza pandemics. The scalable production of mRNA vaccines also facilitates ease of updates to component vaccine strains in response to antigenic drift^13^. The potential advantages of influenza mRNA vaccines targeting HA have been long realized, with preclinical results published as early as 2001, in which Fleeton et al. found two 10 μg doses of mRNA encoding HA from A/Puerto Rico/8/1934 (PR8) was able to protect 90% of mice from subsequent homologous challenge^14^. Subsequently, mice vaccinated with two 80 μg doses of PR8 HA mRNA were shown to have 100% survival following challenge with 10MLD_50_ PR8, and 100% survival with moderate clinical disease when vaccinated with a single 80 μg dose^15^. In 2018 a trivalent self-amplifying mRNA vaccine against HA from H1N1, H3N2, and influenza B viruses was found to be potently immunogenic and protective when given at a dose of 1.25 μg for immunization. This was the first study to demonstrate protective efficacy of a trivalent mRNA vaccine encoding HA from multiple subtypes following both H1N1 and H3N2 challenges^16^. mRNA vaccines for SARS-CoV-2 have been shown to be safe and effective in the clinic, strengthening the viability of this platform for influenza^17,18^. The results of these studies coupled with the success of SARS-CoV-2 mRNA vaccines has spurred the advancement of HA-specific monovalent and quadrivalent mRNA vaccines into clinical trials^19–21^.

The mRNA platform is a powerful development in vaccine technology, not only in regards to their flexibility, speed, and scalability, but in that they offer an alternative approach to the development of a universal influenza vaccine. To that end, in a 2022 paper by Arevalo et al., vaccination with mRNA encoding 20 different HAs, representative of all A and B subtypes, induced humoral responses against all antigens and protected mice and ferrets from influenza challenge with matched and mismatched strains^22^. Furthermore, a natural extension of immunization with multivalent HA mRNA vaccines are multi-antigenic mRNA vaccines. In 2020 Freyn et al. reported broad protection in mice vaccinated with modified mRNA encoding conserved sequences from HA, NA, M2, and NP on a single strand of mRNA at a dose as low as 50 ng^23^. A recent study of a pentavalent modified mRNA vaccine targeting HA, NA, NP, and matrix protein 2 (M2) from B/Victoria/2/1987-like lineage and HA from B/Yamagata/16/1988-like lineage was found to induce broadly cross-reactive antibodies against ancestral and contemporary B viruses from both lineages, as well as protect mice from morbidity and mortality following challenge with a panel of influenza B viruses^24^. Similarly, a 2022 study of mRNA vaccines containing HA, NA, NP, and M2 targeting group 2 influenza viruses was found to protect mice from all challenge viruses, even when administered as a single dose of 125 ng^25^. These results demonstrate the promise of mRNA vaccines to improve breadth and efficacy of influenza vaccines.

The studies presented here add to this growing body of work by demonstrating that quadrivalent influenza mRNA vaccines targeting HA from four seasonal subtypes produced by GreenLight Biosciences (GLB) are potently immunogenic and protective following challenge in the mouse model. Previous studies are expanded upon through preclinical optimization of these vaccines. Such optimization includes protective efficacy studies of modified and unmodified mRNA vaccines, evaluation of immune dampening effects when multiple antigens are concurrently targeted, and method of quadrivalent mRNA-LNP delivery. When assessed in mice, both monovalent and quadrivalent mRNA vaccines encoding HA elicited robust anti-HA antibody titers that were protective against viral challenge. Quadrivalent mRNA vaccines could be administered in formulations in which all mRNA strands were encapsulated in a single LNP or in individual LNPs at doses as low as 0.1 μg while remaining protective against viral challenge. Finally, a single dose of quadrivalent vaccine protected mice from morbidity and mortality as well as those that received two doses of vaccine following challenge.

## Results

### Transfection of mRNA vaccines result in expression of full-length A/California/07/2009 hemagglutinin in vitro

**Fig. 1:**
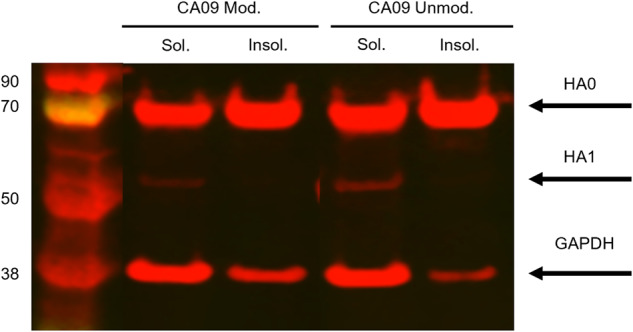
In vitro expression of mRNA encoding A/California/07/2009 hemagglutinin. Protein expression of 293T cells transfected with modified and unmodified mRNA encoding the HA of A/California/07/2009 was analyzed via Western blot; detection of GAPDH was used as a control.

To ensure GLB mRNA vaccines encoding hemagglutinin (HA) could be successfully translated and expressed, 293T cells were transfected with modified and unmodified mRNA vaccines encoding HA from A/California/07/2009. Forty-eight hours after transfection, cells were lysed, and the lysate collected and centrifuged to separate soluble and insoluble fractions. Both fractions were run on MES SDS gels for detection of HA by Western blot. Expression of GAPDH was detected as a control (Fig. 1).

Full-length HA, HA0, is composed of two subunits, HA1 and HA2, that are proteolytically cleaved during entry into the host cell. HA0 has a molecular mass of ~75 kDA, and HA1 and HA2 have molecular masses of ~55 kDa and ~25 kDa, respectively, though sizes can vary between strains due to differences in glycosylation^26^. When CA09 HA expression was evaluated, bands of 70 kDA were observed in both soluble and insoluble fractions, indicating expression of HA0 (Fig. 1). Though semi-quantitative, no appreciable difference in presence of HA0 was observed between either fraction, and both modified and unmodified CA09 mRNA vaccines exhibited similar expression of HA (Fig. 1). Faint bands present above 50 kDa in the soluble fractions indicated low-level expression of HA1 (Fig. 1). Therefore, both modified and unmodified mRNA successfully expressed both full-length and subunit HA in vitro.

### Modified and unmodified mRNA vaccines encoding A/California/07/2009 hemagglutinin protect mice from influenza infection

The first step in evaluating influenza mRNA vaccine efficacy was to determine if use of modified and unmodified nucleosides affected mRNA vaccine immunogenicity or protection during infection. Modified nucleosides, namely pseudouridine, have been well-documented to increase translational efficiency of mRNA vaccines by reducing innate immune signaling and suppressing recognition of foreign dsRNA^27–29^. Furthermore, by dampening the innate immune response the reactogenic profile of mRNA vaccines can be tempered^30–32^. Therefore, to evaluate differences in immunogenicity and protection between mRNA influenza vaccine containing modified and unmodified nucleosides, mRNA encoding CA09 HA was administered to mice at doses of 5 and 30 μg at days 0 and 21. Control mice were given saline or LNP containing mRNA encoding firefly luciferase. Intermittent bleeds were taken for antibody analysis using the hemagglutination inhibition assay (HI). At day 42 (21 days post-boost), mice were challenged with 12MLD_50_ of CA09.

**Fig. 2:**
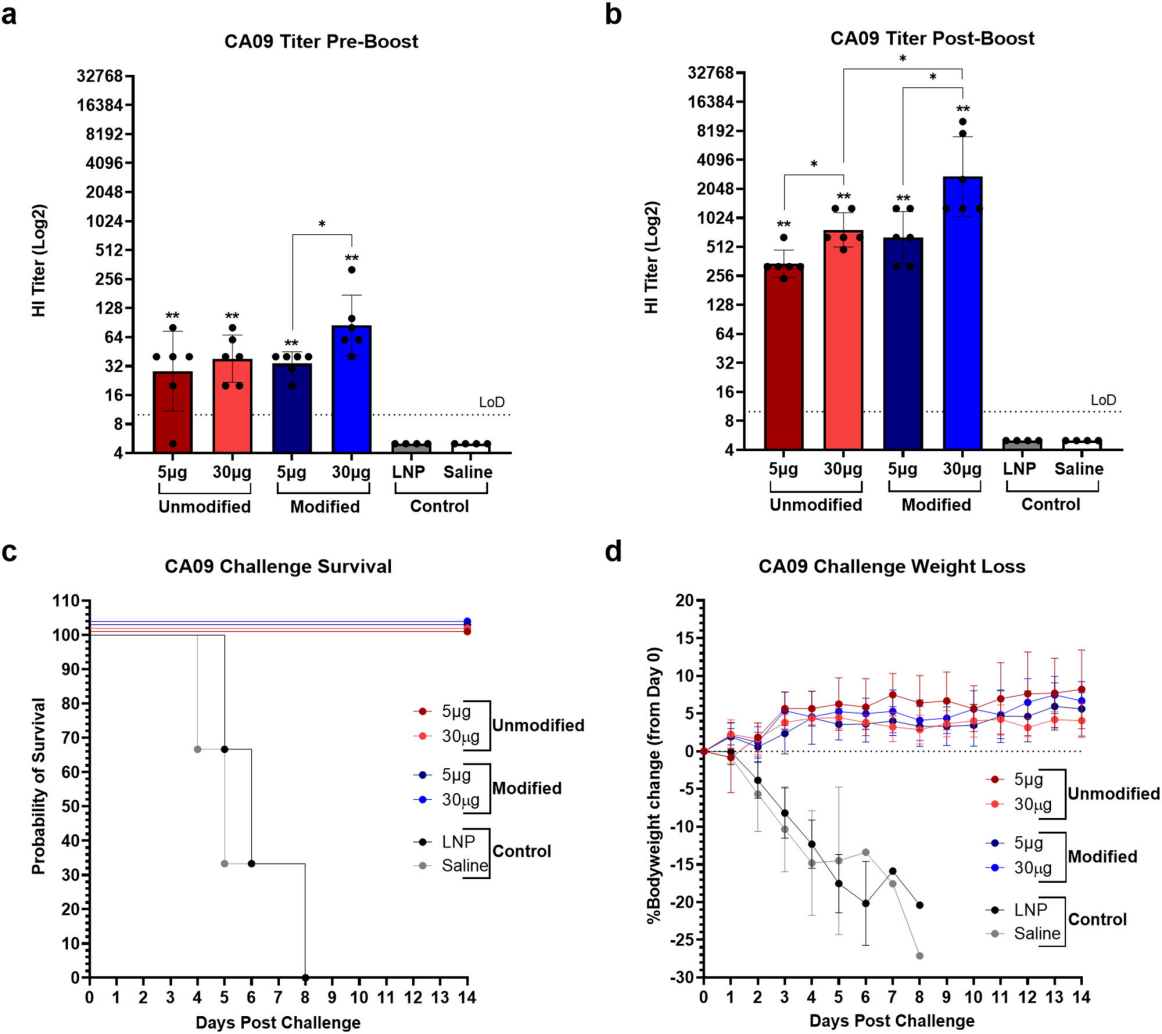
Immunogenicity and protection of modified and unmodified A/California/07/2009 HA mRNA vaccines. 6–8-week-old mice were vaccinated with 5 or 30 µg of modified or unmodified mRNA encoding A/California/07/2009 on days 0 and 21 for antibody analysis. LNP and saline were used as controls. Bleeds were taken at days 0 (pre-boost) and 42 (post-boost). Mice were challenged with A/California/04/2009 at day 42. **a** Anti-HA antibody titers of each vaccine group pre-boost. **b** Anti-HA antibody titers of each vaccine group post-boost. **c** Kaplan–Meier survival curve of each vaccine group following CA09 challenge. **d** Percent bodyweight loss of each vaccine group following CA09 challenge. Error bars represent standard deviation. Statistical analyses were performed using rank-based Mann–Whitney tests with Holm- Šidάk for multiple comparisons. LoD limit of detection; *p < 0.05; **p < 0.01; ***p < 0.001; ****p < 0.0001.

Pre-boost (day 21), anti-HA antibody titers in all vaccine groups were significantly higher than in controls with geometric mean titers (GMTs) of 28.3 and 38.1 in the 5 and 30 μg unmodified groups, respectively, and 34 and 84.7 in the 5 and 30 μg modified groups, respectively (Fig. 2a). The difference in GMT HI titer between the 5 and 30 μg unmodified groups was not significant, but mice that received 30 μg of modified mRNA had significantly higher antibody titers than those that received 5 μg of modified mRNA (Fig. 2a). No statistically significant differences in titer between equivalent doses of each vaccine were found. Pre-challenge and post-boost (day 42), titers in all vaccine groups rose to GMTs of 342.4 and 768.6 in the 5 and 30 μg unmodified groups, respectively, and 640 and 2739 in the 5 and 30 μg modified groups, respectively (Fig. 2b). At this time point, titers in mice that received 30 μg of either mRNA were significantly higher than in the corresponding 5 μg groups (Fig. 2b). Furthermore, antibody induction in mice that received 30 μg of modified mRNA was significantly higher than in those that received 30 μg of unmodified mRNA (Fig. 2b). The difference in titer between the 5 μg groups was not statistically significant. Following challenge, all mice that received CA09 HA mRNA were completely protected from challenge with no observed clinical signs or weight loss (Fig. 2c, d).

### Quadrivalent seasonal hemagglutinin mRNA vaccination induces protective antibody titers against all subtypes comparable to monovalent vaccination

As currently licensed influenza vaccines target HA from representative strains of three or four subtypes/types of seasonal influenza viruses, the immunogenicity and efficacy of a quadrivalent mRNA vaccine that encodes the HA of CA09, A/Hong Kong/4801/2014 H3N2 (HK14), B/Brisbane/02/2008 B-Victoria lineage (BBris), and B/Phuket/3073/2013 B-Yamagata lineage (BPhu) was next assessed. Mice were vaccinated with 30 μg of mRNA for each antigen individually or quadrivalently (QmRNA; 30 μg of each for 120 μg total) on a prime-boost schedule 21 days apart. Control animals were again given monovalent vaccine, saline, or LNPs containing irrelevant mRNA on the same prime-boost regimen and anti-HA antibody titers were determined by HI. Mice were challenged with 12MLD_50_ of CA09 21 days post-boost.

**Fig. 3:**
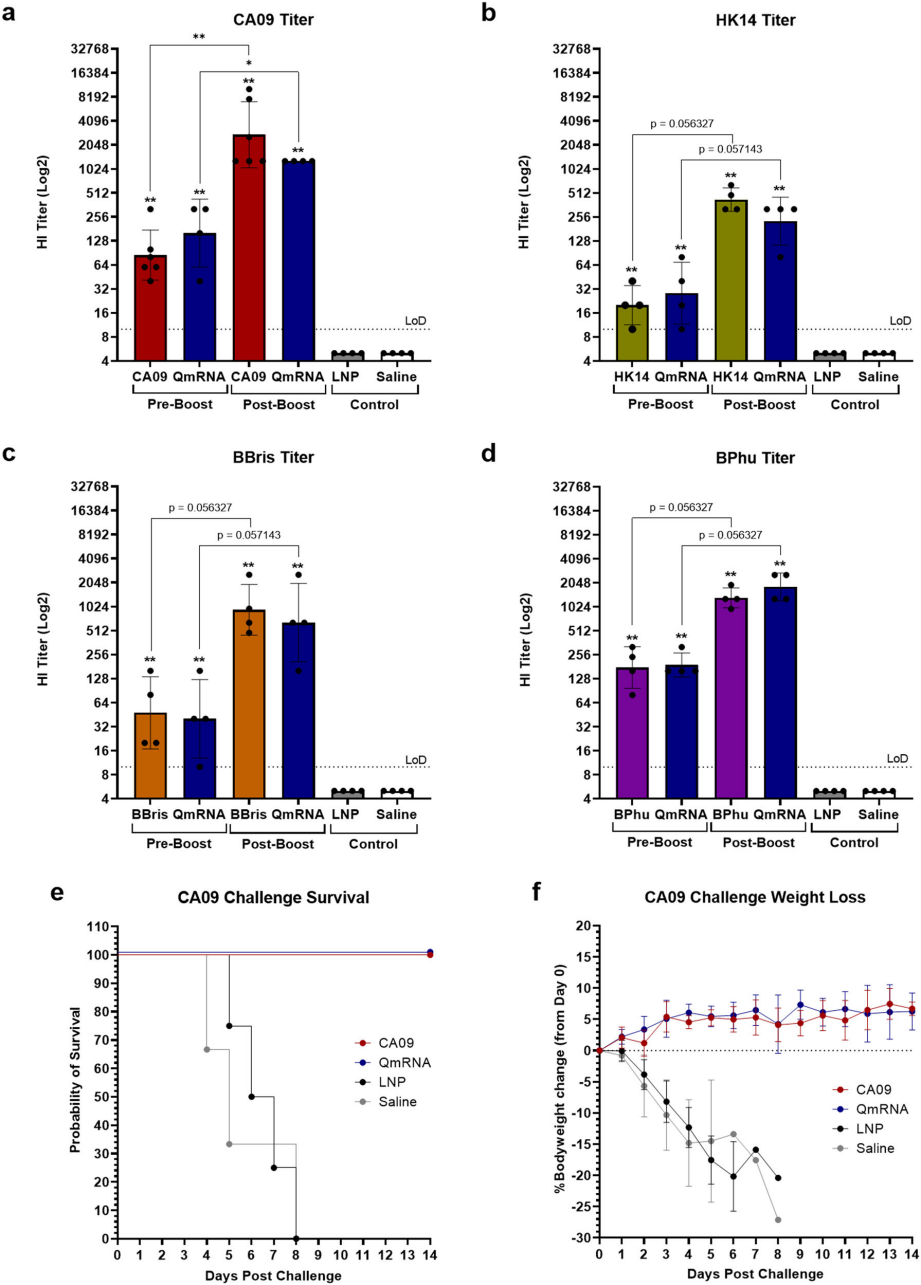
Immunogenicity and protection of monovalent vs. quadrivalent HA mRNA vaccines. 6–8-week-old mice were vaccinated with 30 µg of monovalent seasonal HA mRNA or quadrivalent seasonal HA mRNA (120 µg total) on days 0 and 21. LNP and saline were used as controls. Bleeds were taken at days 0 (pre-boost) and 42 (post-boost) for antibody analysis. Mice were challenged with A/California/04/2009 at day 42. **a** CA09 anti-HA antibody titers of each vaccine group pre- and post-boost. **b** HK14 anti-HA antibody titers of each vaccine group pre- and post-boost. **c** BBris anti-HA antibody titers of each vaccine group pre- and post-boost. **d** BPhu anti-HA antibody titers of each vaccine group pre- and post-boost. **e** Kaplan–Meier survival curve of CA09 and quadrivalent vaccine groups following CA09 challenge. **f** Percent bodyweight loss of CA09 and quadrivalent vaccine groups following CA09 challenge. Error bars represent standard deviation. Statistical analyses were performed using rank-based Mann–Whitney tests with Holm–Šidάk for multiple comparisons. LoD limit of detection; *p < 0.05; **p < 0.01; ***p < 0.001; ****p < 0.0001.

Pre-boost (day 21), vaccine groups exhibited anti-HA antibody induction against all antigens that were significant compared to saline and LNP control animals, with GMTs of 84.7 and 160 in monovalent and quadrivalent groups when tested against CA09, respectively, 20 and 28.3 in monovalent and quadrivalent groups when tested against HK14, respectively, 47.6 and 40 in monovalent and quadrivalent groups when tested against BBris, respectively, and 177.1 and 190.3 in monovalent and quadrivalent groups when tested against BPhu, respectively (Fig. 3a–d). Post-boost (day 42), HI titers rose against all antigens with GMTs of 2739 and 1280 for CA09 monovalent and quadrivalent groups, respectively, 421.1 and 226.3 for HK14 monovalent and quadrivalent groups, respectively, 932.1 and 640 for BBris monovalent and quadrivalent groups, respectively, and 1318.3 and 1810.2 for BPhu monovalent and quadrivalent groups, respectively (Fig. 3a–d). Post-boost rises in titer compared to pre-boost titers were statistically significant against CA09 but not in the other antigen groups (Fig. 3a). At no time point were significant differences in antibody titer observed between monovalent and quadrivalent groups for any antigen, suggesting the administration of multiple mRNAs did not dampen the humoral response to individual antigens within the vaccine. Following challenge, mice that received monovalent CA09 or quadrivalent vaccine were completely protected from challenge, exhibiting 100% survival and no bodyweight loss (Fig. 3e, f). Therefore, mRNA vaccines for influenza can be administered in quadrivalent formulations to induce robust, protective anti-HA antibody responses against multiple viruses that are protective following CA09 challenge.

### Admixed and co-formulated quadrivalent seasonal hemagglutinin mRNA vaccination protects against A/California/04/2009 infection at low doses

The previous experiments were used to establish efficacy of the mRNA vaccine platform, but high doses of the vaccine limited nuanced evaluation of dose or subtype differences. Additionally, the above quadrivalent study used co-formulated mRNA vaccine; that is, mRNA for each antigen was encapsulated in a single LNP. Quadrivalent mRNA vaccines may also be “admixed” wherein each LNP contains mRNA encoding an individual antigen; mRNA-LNP complexes are then mixed prior to injection. To detect differences in antibody induction between each subtype and formulation, mice were vaccinated with quadrivalent mRNA at doses ranging from 10 to 0.01 μg of each antigen (40–0.04 μg total mRNA) at days 0 and 21; 21 days post-boost, mice were challenged with 12MLD_50_ of CA09. In addition, positive control mice were given two doses of 1.5 µg of 2017–2018 inactivated quadrivalent influenza vaccine (QIV) at days 0 and 21. Again, negative control animals were given saline or LNPs containing irrelevant mRNA and anti-HA antibody titer determined by HI. Of note, two days after vaccination one animal in the co-formulated 0.1 μg group was found dead. Necropsy of the animal concluded the death was “spontaneous or environmental disease unrelated to vaccine.” This group subsequently had a sample size of 3.

**Fig. 4:**
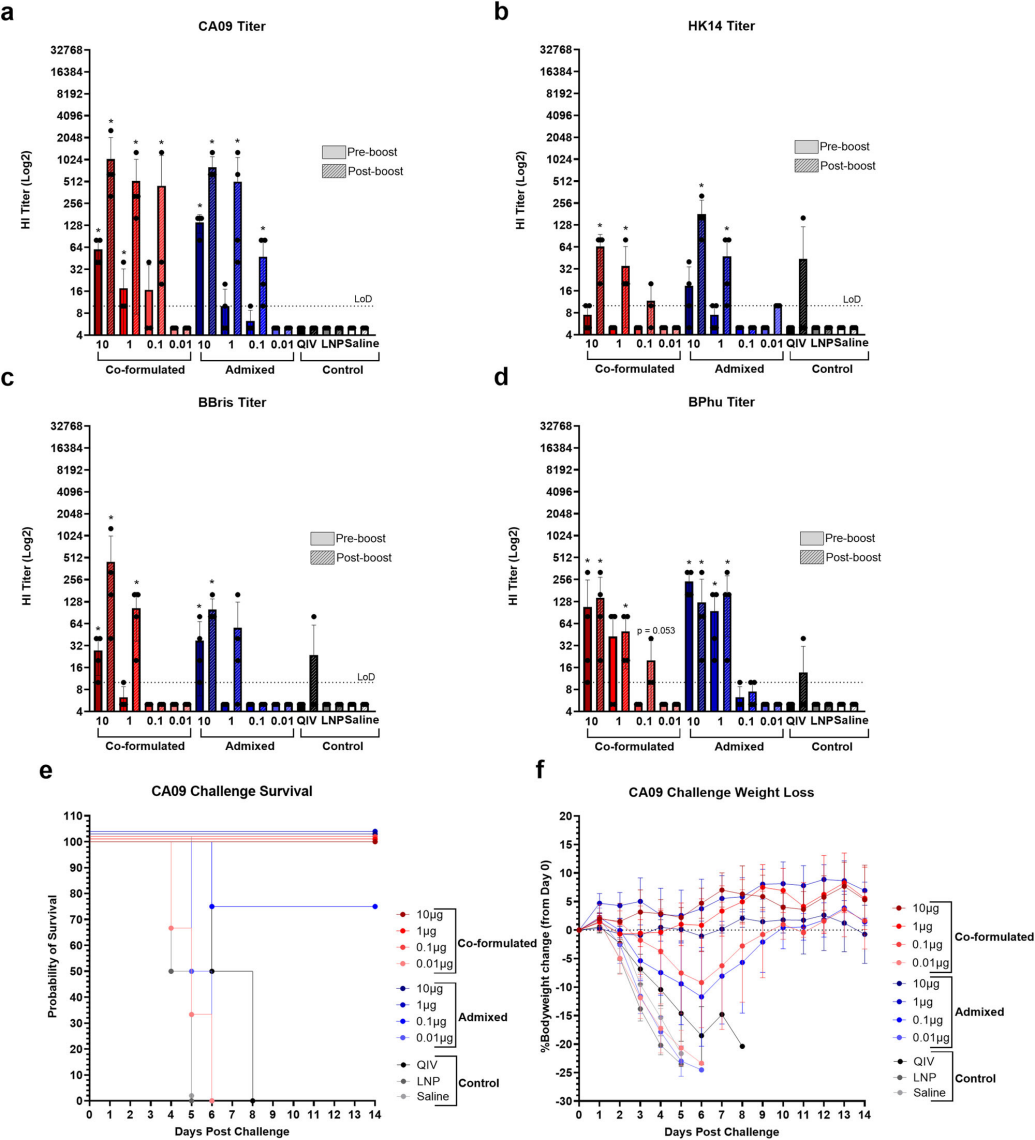
Immunogenicity and protection of co-formulated vs. admixed HA mRNA vaccines. 6–8-week-old mice were vaccinated with quadrivalent vaccine containing 0.01–10 µg of mRNA encoding each HA subtype (0.04–40 µg total) or 1.5 µg of QIV on days 0 and 21. LNP and saline were used as controls. Bleeds were taken at days 0 (pre-boost) and 42 (post-boost) for antibody analysis. Mice were challenged with A/California/04/2009 at day 42. **a** CA09 anti-HA antibody titers of each vaccine group pre- and post-boost. **b** HK14 anti-HA antibody titers of each vaccine group pre- and post-boost. **c** BBris anti-HA antibody titers of each vaccine group pre- and post-boost. **d** BPhu anti-HA antibody titers of each vaccine group pre- and post-boost. **e** Kaplan–Meier survival curve of each vaccine group following CA09 challenge. **f** Percent bodyweight loss of each vaccine group following CA09 challenge. Error bars represent standard deviation. Statistical analyses were performed using rank-based Mann–Whitney tests with Holm–Šidάk for multiple comparisons. LoD limit of detection; *p < 0.05; **p < 0.01; ***p < 0.001; ****p < 0.0001.

Pre-boost (day 21), co-formulated mRNA induced anti-HA antibody titers against CA09 that were significantly elevated compared to control animals at the 10 and 1 μg doses with GMTs of 56.6 and 14.1, respectively, while only the 10 μg dose of admixed vaccine resulted in antibody induction (GMT of 134.5) that was statistically significant compared to the control groups (Fig. 4a). Post-boost (day 42), both co-formulated and admixed mRNA induced HI titers against CA09 that were significant compared to control animals at the 10, 1, and 0.1 μg doses with co-formulated GMTs of 761.1, 380.5, and 100.8, respectively, and admixed GMTs of 761.1, 226.274, and 33.6, respectively (Fig. 4a). When antibodies against HK14 were assayed post-boost, both co-formulated and admixed vaccines given at 10 and 1 μg induced titers that were significantly higher than controls with co-formulated GMTs of 56.6 and 28.3, respectively, and admixed GMTs of 160 and 33.6, respectively (Fig. 4b). When anti-HA BBris antibodies were evaluated, mice that received 10 μg of co-formulated vaccine had significantly higher titers (GMT of 23.8) than controls pre-boost. Post-boost, these titers rose in mice that received 10 or 1 μg (GMTs of 226.3 and 80, respectively) to statistical significance. Pre- and post-boost titers in those that received 10 μg of admixed vaccine (GMTs of 28.3 and 95.1, respectively) were also significant compared to controls (Fig. 4c). When antibody titers against BPhu were evaluated, mice that received 10 μg of vaccine had titers that were significant compared to saline and LNP controls both pre- and post-boost with GMTs of 47.6 and 95.1, respectively, in the co-formulated groups and 226.3 and 80, respectively, in the admixed groups (Fig. 4d). Additionally, titers in mice that received 1 μg of admixed vaccine were also significant pre- and post-boost (GMTs of 67.3 and 113.1, respectively). Titers in mice that received co-formulated vaccine at this dose became statistically significant following boost (pre- and post-boost GMTs of 20 and 40, respectively). No statistically significant differences in titer between pre- and post-boost were found in either formulation against any antigen at any dose (Fig. 4a–d). Modest dose-response effects were observed for each antigen, though no significant differences in antibody titer between doses within admixed and co-formulated groups, or between equivalent doses of each formulation were found either pre- or post-boost (Fig. 4a–d). Of note, only two mice developed HI titers against the evaluated strains when QIV was administered. Anti-HA antibody titers were observed against HK14 and BPhu in the first animal, while HI titers against BBris were present in the second (Fig. 4a–d). Therefore, both admixed and co-formulated quadrivalent mRNA vaccines more effectively induced anti-HA antibodies against all targeted strains when given at a comparable dose of 1 µg.

Dose and formulation differences in antibody induction against CA09 were reflected following challenge as mice that received 10 or 1 μg of co-formulated and admixed vaccine had 100% survival with no observed morbidity (Fig. 4e, f). In contrast, all mice that received 1.5 µg of QIV exhibited pronounced weight loss and succumbed to infection (Fig. 4e, f). Mice that received 0.1 μg of co-formulated vaccine also had 100% survival but lost up to ~8% bodyweight (Fig. 4e, f). Similarly, mice vaccinated with 0.1 μg of admixed vaccine had 75% survival with modest bodyweight loss of ~10% (Fig. 4e, f). Mice that were given 0.01 μg of either formulation had a 0% survival rate (Fig. 4e, f). The results of this study demonstrate that quadrivalent mRNA vaccines given at low doses induce differential antibody responses between subtypes that correspond to reductions in protection from infection as antibody titers decline. As no appreciable differences in immunogenicity and protection between co-formulated and admixed vaccines were found, and because the admixed formulation strategy provides more flexibility when manufacturing multi-component vaccines, the admixed formulation was used in all subsequent studies.

### Quadrivalent seasonal hemagglutinin mRNA vaccination protects against A/California/04/2009 infection following a single dose

**Fig. 5:**
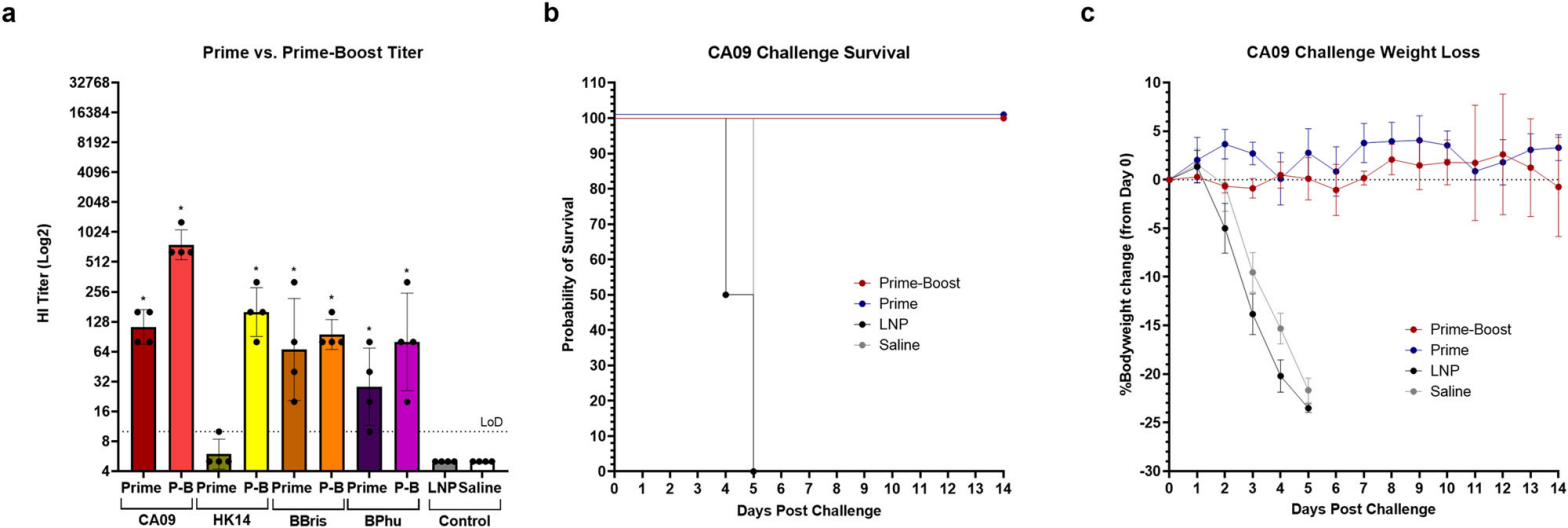
Immunogenicity and protection of 1 vs. 2 doses of HA mRNA vaccine. 6–8-week-old mice were vaccinated with quadrivalent vaccine containing 10 µg of mRNA encoding each HA subtype (40 µg total) on day 0 (prime) or days 0 and 21 (prime-boost). LNP and saline were used as controls. Bleeds were taken at days 0 (pre-boost) and 42 (post-boost) for antibody analysis. Mice were challenged with A/California/04/2009 at day 42. **a** Anti-HA antibody titers against each antigen following one or two doses of mRNA vaccine. **b** Kaplan–Meier survival curve of each vaccine group following CA09 challenge. **c** Percent bodyweight loss of each vaccine group following CA09 challenge. Error bars represent standard deviation. Statistical analyses were performed using rank-based Mann–Whitney tests with Holm- Šidάk for multiple comparisons. LoD limit of detection; P-B prime-boost; *p < 0.05; **p < 0.01; ***p < 0.001; ****p < 0.0001.

As seasonal influenza vaccines are administered annually as a single dose in adults, the immunogenicity and efficacy of a single dose of admixed quadrivalent mRNA vaccine was next evaluated. Mice received 10ug of quadrivalent mRNA encoding each antigen (40 μg total) at day 0 (Prime) or days 0 and 21 (P-B) and, again, challenged with 12MLD_50_ of CA09 42 days post-initial immunization. When antibody induction was evaluated by HI in mice that received two doses of vaccine, anti-HA antibody titers were significant for all antigens when compared to LNP and saline controls with GMTs of 761.1, 160, 95.1, and 80 against CA09, HK14, BBris, and BPhu, respectively (Fig. 5a). At day 42 in mice that received a single dose, antibody titers against CA09, BBris, and BPhu were significant compared to controls with GMTs of 113.1, 67.3, and 28.3, respectively; however, a single 10ug dose of vaccine resulted in poor antibody induction (GMT of 5.9) against HK14 (Fig. 5a). There were no statistically significant differences in titer between one and two doses for any antigen; administration of a second dose did result in markedly higher titers against HK14, as discussed above, but did not reach statistical significance (Fig. 5a). Following challenge, mice that received either one or two doses of vaccine were completely protected from CA09 challenge, exhibiting no morbidity, and having 100% survival (Fig. 5b, c). Therefore, administration of a single dose of quadrivalent influenza mRNA vaccine resulted in robust antibody induction against most antigens that were protective following CA09 challenge.

## Discussion

Preclinical studies of influenza mRNA vaccines targeting HA have been ongoing since as early as 2001 and have shown robust antibody induction and protection from challenge in a variety of animal models for both seasonal and pandemic subtypes^14,15,33–35^. The studies presented here add to this growing body of work by demonstrating that quadrivalent influenza mRNA vaccines targeting HA from four seasonal subtypes are potently immunogenic and protective following challenge in the mouse model and can be optimized to ensure protective efficacy while enabling flexible manufacture.

mRNA construction was first optimized by evaluating immunogenicity and protective efficacy of modified and unmodified CA09 HA mRNA vaccines. Though both mRNA vaccines provided robust protection from viral challenge, modified mRNA induced significantly higher antibody titers than unmodified mRNA in the high-dose groups post-boost. Additionally, use of pseudouridine is consistent with mRNA vaccines currently available in the clinic, as most notably employed by both Moderna and Pfizer in the generation of their SARS-CoV-2 mRNA vaccines which both employ 1-methyl-pseudouridine^36,37^. Therefore, based on the results of this study and demonstrated safety and efficacy in the clinic, we chose to pursue modified nucleoside mRNA vaccines.

To ensure immune activation, we first administered monovalent and quadrivalent mRNA at doses of 30 μg of each antigen for a total of 120 μg in the quadrivalent group. This strategy resulted in antibody induction against each subtype that was consistent between monovalent and quadrivalent groups, demonstrating that potency of the mRNA vaccine is preserved when multiple antigens are targeted. Though no adverse reactions were observed in the mice, a total concentration of 120 µg of mRNA vaccine is higher than Spikevax and COMIRNATY adult dosing guidelines of two administrations of 100 and 30 μg, respectively^17,18^. Therefore, we next evaluated the protective efficacy of the quadrivalent mRNA vaccine when given at total doses of 40–0.04 µg. Though no clear dosing guidelines for influenza mRNA vaccines are available for humans at this time, early results from the phase I trial of Moderna’s quadrivalent seasonal flu vaccine, mRNA 1010, found that 50 µg was sufficient to induce GMTs of 538, 530, 467, and 261 against H1N1, H3N2, B-Victoria, and B-Yamagata strains, respectively, in young adults, and GMTs of 310, 263, 305, and 215 against H1N1, H3N2, B-Victoria, and B-Yamagata strains, respectively, in adults ≥50^38^. These results suggest that a dose of 40 µg of each strain for quadrivalent mRNA, as evaluated here, may be sufficient for clinical use.

When quadrivalent vaccine dose was lowered, immunogenicity was maintained, and even at 1 μg of each component, quadrivalent mRNA vaccines protected against A/California/04/2009 challenge with little to no observed morbidity, whereas those that received a similar amount of QIV were not protected from influenza infection. In contrast, a study by Groves et al. found that mice given 1.5 μg of HA protein were only partially protected from A/California/07/2009 challenge, losing up to 15% bodyweight^39^. The low doses of quadrivalent vaccine evaluated here were effective regardless of mRNA-LNP administration method; however, when considering manufacturing constraints and the need to update component vaccine strains annually, the admixed formulation may lend itself more easily to these considerations. The results of the admixed dose de-escalation were also consistent with a study by Kistner et al. that found two 3.75 μg doses of whole inactivated CA09 provided 100% protection following CA09 challenge while two 0.15 μg doses resulted in 80% protection from CA09 challenge^40^. Finally, administration of a single 10 μg dose of GLB quadrivalent mRNA fully protected mice from CA09 challenge, though antibody induction against the H3N2 HK14 antigen after a single dose at this concentration was poor. It is unclear why immunization against HK14 HA resulted in substantially lower antibody induction following a single administration than the other antigens evaluated within these studies. Vaccination against H3N2 with recombinant protein or inactivated trivalent vaccine has been previously documented to induce lower HI titers than H1N1 so this may be an intrinsic feature of the H3 protein^41^. Though not used in the construction of these GLB mRNA vaccines, codon optimization of DNA vaccines has been found to improve H1 and H5 immunogenicity and may be an effective strategy to improve the potency of the H3 mRNA^42,43^. Larger doses of quadrivalent mRNA may also be considered. Alternatively, quadrivalent vaccines in the admixed formulation could be composed of varying concentrations of mRNA to optimize antibody induction against each antigen. This method is currently untested and will require further study to ensure each antigen remains appropriately immunogenic.

The results presented here are encouraging and support the effectiveness of GLB quadrivalent influenza mRNA vaccines in generating HA-targeted neutralizing antibody responses which have been well-documented to correlate with protection from disease^44–46^. In addition to antibodies targeting HA, T cells are also an important immune mediator of infection and deserve analyses in future studies^47^. The presence of pre-existing CD4^+^ T cells has been shown to correlate with protection from influenza disease in humans, while memory CD8^+^ T cell activity is associated with protection from emerging influenza viruses^48–50^. An early study of influenza mRNA vaccines by Petsch et al. found mice vaccinated with PR8 HA mRNA exhibited CD4^+^ responses against a variety of MHC class II PR8 peptides as well as greater CD8^+^ cytotoxic activity than controls when tested with MHC class I PR8 peptides^15^. A study published by Chivukula et al. in 2021 found unmodified mRNA vaccines encoding H1N1 or H3N2 HA and NA induced humoral and cellular responses in mice that were protective following challenge^51^. Further, in nonhuman primates HA-stalk specific and NP mRNA vaccines have been found to elicit robust anti-stalk antibodies following HA immunization and CD4^+^ and CD8^+^ T cell responses when vaccinated with NP mRNA; individually, both vaccines partially protected mice from lethal influenza challenge and were efficacious when administered together^52^. T cell induction has also been well-documented following SARS-CoV-2 mRNA vaccination; preclinical evaluation of the Pfizer BNT162b SARS-CoV-2 mRNA vaccine has demonstrated induction of Th1-biased CD4^+^, CD8^+^, and T_FH_ responses in both mice and rhesus macaques, as did vaccination of rhesus macaques with Moderna mRNA-1273, though little CD8^+^ activity was observed after mRNA-1273 immunization^53,54^. Consistent with these findings, we have found that SARS-CoV-2 mRNA vaccines produced by GreenLight Biosciences induce Th1-biased CD4^+^ and CD8^+^ T cell responses in mice^55^. Further investigation is needed to fully elucidate the effect of influenza mRNA vaccination on T cell response, and future studies of GLB quadrivalent mRNA vaccines should include this analysis.

Another marker of vaccine potency and efficacy that should be evaluated in subsequent quadrivalent influenza mRNA vaccine studies is longevity of the antibody response. Decline of IIV efficacy over time has been well-documented, even over the course of a single influenza season and is thought to correspond to waning antibody titers^56,57^. This decline is particularly pronounced in the elderly population that struggles to mount and maintain robust antibody responses to immunization with IIV and is at higher risk of influenza-related disease. Age has been found to be associated with early decline of HI titers to non-protective levels six months post-vaccination while a meta-analysis of studies reporting longitudinal HI titers found that by one year post-vaccination, titers fell to pre-vaccination levels in older adults^58,59^. Clinical data on the longevity of influenza mRNA vaccines is not currently available, but SARS-CoV-2 mRNA vaccination has been shown to result in antibody titers that peak 3–4 weeks post-boost and begin to significantly decline 4–6 months later^60^. Future quadrivalent mRNA studies should include longitudinal serum sampling to understand the kinetics of antibody maintenance post-vaccination.

All studies presented here were conducted in the murine model and while mice have several attractive features for use in the lab, the mouse model of influenza infection has notable weaknesses. As most influenza viruses do not cause natural disease in mice, influenza viruses must be adapted for murine infection, limiting the availability of challenge strains^61,62^. This limitation was reflected in the challenge data presented here; though mice were vaccinated with HA for multiple influenza subtypes, only CA09 was used for challenge as other mouse adapted virus stocks were not available. Therefore, protective efficacy of a quadrivalent mRNA vaccine could not be evaluated against seasonal influenza subtypes other than H1N1 and will have to be addressed in the future either through generation of mouse adapted viruses or with a different animal model.

Overall, when preclinically assessed in mice, administration of quadrivalent mRNA vaccines targeting HA from four seasonal influenza subtypes resulted in robust antibody induction and protection from challenge in both mono and quadrivalent formulations. Admixed and co-formulated mRNA-LNP quadrivalent vaccines were both highly effective when administered at doses as low as 1 μg, remaining robustly immunogenic and protective against A/California/04/2009 infection, though use of the admixed formulation more easily enables customization of individual mRNAs and updates to component strains. Finally, administration of a single 10 μg dose of admixed quadrivalent mRNA-LNP induced antibody titers that were fully protective following A/California/04/2009 challenge. The results reported here demonstrate the potent immunogenicity and protective efficacy of quadrivalent mRNA-LNP influenza vaccines in a preclinical model.

## Methods

### mRNA vaccine preparation

mRNA vaccines were manufactured by GreenLight Biosciences. mRNA constructs contained modified (pseudouridine) or unmodified nucleosides and were non-stabilized into prefusion states. Sequences used for mRNA production were taken from GISAID and are as follows: A/California/07/2009 HA (EPI177294), A/Hong Kong/4801/2014 HA (EPI539576), B/Brisbane/60/2008 HA (EPI394898), and B/Phuket/3073/2013 HA (EPI529345). mRNA vaccines were encapsulated in lipid nanoparticles by Acuitas Therapeutics. The LNP is composed of 4 lipid components: a proprietary cationic lipid, a saturated phospholipid, a PEG-lipid and cholesterol. Admixed quadrivalent vaccines were created by mixing the fully formulated mRNA-LNPs. mRNA vaccines were diluted to final doses in sterile saline (Hospira 0409-4888-02).

### mRNA transfection

293T cells were plated the day before transfection in 24-well flat bottom tissue culture treated plates, to be near 80%+ confluence the following day in MEM (Corning 10-010-CV) + 1% vitamin solution (Gibco 1112-052) + 1% antibiotic/antimycotic (Gibco 15240-062) + 1% L-Glutamine (Gibco 25030-081). To transfect, un-supplemented MEM was warmed at 37 °C before addition of 1.5 μL of MessengerMax Lipofectamine (Thermo Fisher LMRNA001) per 25 μL MEM and incubated at room temperature for 10 min. During the incubation, 1 μL of 1 mg/mL mRNA stock was added to 50 μL of MEM. 25 μL of mRNA+MEM was added to 25 μL of MEM+Lipofectamine to obtain a 1:1 solution and incubated at room temperature for 10 min. Following incubation, the mRNA solution was added to one well/mRNA of the seeded 24-well plate and incubated for 48 h at 37 °C, 5% CO_2_. After the 48-h incubation, lysates were collected by adding 100 μL of Lysis Buffer (Promega E194A) diluted to 1X in PBS to each well; lysate was removed and placed in labeled Eppendorf tubes. To separate soluble and insoluble fractions, lysate was spun at 15,871 × *g* for 1 min. The soluble fraction was placed in a clean Eppendorf and stored at −20 °C until needed. The insoluble fraction was resuspended in 100 μL of 1X lysis buffer and stored at −20 °C until needed.

### Western blot

Western blot gels were run using the NuPAGE MES kit (ThermoFisher NP0060). First, samples were prepared by combining 2.5 μL loading dye, 1 μL reducing agent, 2.5 μL Lysis Buffer (Promega E194A) diluted to 1X in PBS, and 4 μL of soluble or insoluble lysate from the mRNA transfections. Samples were then heated at 95 °C for 5 min. To prepare running buffer, 50 mL of 20X running buffer was added to 950 mL of dH_2_O and 2.5 mL of antioxidant. The gel cassette was prepared by assembling with tank apparatus; the comb was removed from the gel and wells were rinsed 3x with running buffer before being placed in the tank. Running buffer was added to the tank until the conductive wire was covered. 10 μL of each prepared sample was added to the wells with a 1 kDa protein ladder (Li-Cor 926-98000) for reference. The gel was run for 45 min at 150 V.

Next, the blot was transferred to a nitrocellulose membrane using the BioRad Trans-Blot Turbo Transfer System and associated reagents (BioRad 17001915). To transfer, the gel and nitrocellulose membrane was placed in a cassette according to the manufacturer’s instructions. The blot was transferred using the mixed molecular weight setting for 7 min. Following transfer, the blot was blocked overnight at 4 °C with rocking in blocking buffer (Li-Cor 927-60001).

After blocking, the blocking buffer was removed and 7 mL of primary antibody (A/California/07/2009 anti-HA rabbit pAb (Sino Biologicals 11085-T54), A/Hong Kong/4801/2014 anti-HA mouse mAb (eEnzyme MIA-H3-HK014), B/Brisbane/60/2008 anti-HA mouse mAb (eEnzyme MIB-HA-324), B/Phuket/3073/2013 anti-HA mouse mAb (eEnzyme MIB-HA-P12)) diluted 1:2000 in wash buffer (PBS + 1% Tween-20) was added to the blot and incubated for 2 h at room temperature with rocking. GAPDH was detected as a loading control using anti-GAPDH mouse mAb (Cell Signaling Technology 97166). After the incubation, the primary antibody was removed and the blot washed 5x with 7 mL of wash buffer, rocking for 5 min at room temperature during each wash. The blot was then covered with 8 mL of fluorescently labeled secondary antibody (goat anti-mouse IgG Li-Cor 926-32210; goat anti-rabbit IgG Li-Cor 926-68071) at a 1:10,000 dilution and incubated for 1 h at room temperature with rocking. Following the secondary antibody incubation, the blot was again washed 5x as described. Finally, the blot was visualized using the Li-Cor Odyssey Imaging System at 600 and 800 nm. Blots were from the same experiment or processed in parallel.

### Influenza virus propagation

Influenza viruses were grown in embryonated hens’ eggs as previously described^63^. Briefly, virus was diluted in sterile PBS + 1% antibiotic stock containing 200,000 units penicillin (Sigma P7794), 40,000 units streptomycin (Sigma S9137), 20,000 units Polymixin B (Fresenius Kabi 320110), and 4 mg gentamicin (Fresenius Kabi 1002). 100 μL of virus dilution was injected into the allantoic cavity with 1 mL syringes fitted with 25-gauge × 5/8-inch needles (Exel 26046). Eggs injected with A and B subtypes were incubated at 37 °C and 33 °C, respectively, for 48 h and chilled at 4 °C overnight before harvest. Allantoic fluid was removed and hemagglutination assays performed to determine titer. Viruses were sterility tested with blood agar plates (Edge Biologicals 2P-075) and confirmed via sequencing. Aliquots were stored at −80 °C until needed.

### Mouse models

Eight-week-old female C57BL/6 mice (*n* = 4 or 6 per group) were vaccinated with mRNA encoding seasonal influenza hemagglutinin. Mice that were vaccinated with CA09 HA had a sample size of *n* = 6. All other vaccination groups had samples sizes of *n* = 4. Animals received either a prime-only or prime-boost series of one or two intra-muscular injections, at days 0 and 21, containing a variety of mRNA concentrations in a volume of 50 μL per hind limb. Positive control mice received two 1.5 µg doses of 2017–2018 licensed inactivated quadrivalent influenza vaccine (*Fluzone;* Sanofi Pasteur inc. 49281-417-88). Negative control animals received 50 μL of saline or LNP-encapsulated firefly luciferase mRNA in the hind limb at days 0 and 21. Serum samples were collected at days 0, 21, and 42. Forty-two days after the priming dose, 20 μL of 1 × 10^4.67^ TCID_50_/mL mouse adapted A/California/04/2009 was administered intranasally to isoflurane-anesthetized animals. Following challenge, mice were weighed daily for 14 days. A humane endpoint of ≥20% bodyweight loss was used. Animal studies were conducted in accordance with the Guide for the Care and Use of Laboratory Animals of the National Institutes of Health and approved under St. Jude Children’s Research Hospital’s Animal Care and Use Committee protocol 442.

### Hemagglutination inhibition assay

Hemagglutination inhibition assays were performed as previously described^63^. Prior to assay, sera were RDE treated by adding 3x volume of receptor destroying enzyme (Accurate Chemical YCC340122) to 1x volume of sera and incubated at 37 °C overnight. Sera was then heat treated at 56 °C for 1 h after which 6x volume of PBS was added to obtain a 1:10 starting dilution of sera. Sera was serially diluted in 25 μl of PBS in 96-well u-bottom microtiter plates and incubated with 25 μl of virus standardized to four agglutinating doses for 1 h at room temperature. 50 μl of guinea pig red blood cell suspension (Rockland R305-0050) at 1% in PBS + 0.5% BSA (Sigma A8327) was added to each well and the plates were incubated at room temperature for 1 h. Antibody titer was calculated as the inverse of the highest serum dilution that prevented agglutination. Samples that scored below the dilution threshold of 1:10 were given a value of 5.

### Statistics

Statistical tests were performed with GraphPad Prism Software, including Kaplan–Meier survival curves. Saline and LNP control values were combined for statistical analysis. Rank-based Mann–Whitney was used for statistical analysis and corrected for multiple comparisons by the Holm–Šidάk method.

### Reporting summary

Further information on research design is available in the Nature Research Reporting Summary linked to this article.

### Supplementary information

REPORTING SUMMARY

## Data Availability

Primary data sets are available upon request.
